# Operational Efficiency of Public Hospitals in Greece During the COVID-19 Pandemic: A Comparative Analysis Using DEA and AHP Models

**DOI:** 10.3390/jmahp12040030

**Published:** 2024-12-10

**Authors:** Athanasios Mitakos, Panagiotis Mpogiatzidis

**Affiliations:** Department of Midwifery, School of Health Sciences, University of Western Macedonia, 50200 Ptolemaida, Greece; dmw00017@uowm.gr

**Keywords:** COVID-19, hospital efficiency, Data Envelopment Analysis (DEA), Analytic Hierarchy Process (AHP), efficiency during crisis

## Abstract

This study evaluates the efficiency of public hospitals in Greece during the COVID-19 epidemic in 2020, using Data Envelopment Analysis (DEA) and the Analytical Hierarchy Process (AHP). Faced with unprecedented pressure from increased demand for medical services, these hospitals had to adapt quickly while playing a crucial role in supporting local economies, similar to the effect of tourism on rural economies. This study reveals that, despite average efficiency scores of 83% for result-oriented models (BCC) and 65% for constant return models (CCR), inefficiencies of scale emerged under the pressures of the pandemic. The AHP, by incorporating qualitative criteria and decision-makers’ preferences, offers a valuable perspective but shows little correlation with DEA’s quantitative results. This research emphasizes the importance of utilizing integrated methods to formulate a more comprehensive assessment, adapted to the complex challenges of the healthcare sector during crisis periods.

## 1. Introduction

The effectiveness of public hospitals in Greece during the COVID-19 epidemic in 2020 represents a crucial area of study. The role of these hospitals during the pandemic became a focal point due to the increasing demands placed on healthcare systems [1]. Hospitals were under unprecedented pressure from the urgent need for medical services, extended treatment times and the high transmissibility of the virus [2]. This scenario marked a clear divergence from traditional healthcare dynamics and required rapid adaptation on the part of hospital administrations. From an economic point of view, public hospitals were not only health care providers but also key drivers of stability within local economies during the pandemic. The influx of funding for healthcare services and the strategic deployment of resources underlined the role of hospitals in supporting local economies [3]. During this period, public hospitals played a key role not only in managing the direct health impacts of the virus but also in supporting broader entrepreneurial objectives within the healthcare sector. These included the rapid expansion of digital health services and innovations in patient care management [1,3]. Such initiatives improved the overall quality of healthcare delivery and provided an economic cushion against the adverse effects of the pandemic on other sectors.

Addressing the operational challenges and improving the efficiency of these hospitals remains a key concern. In this context, efficiency is defined as the degree of operational excellence achieved through the rational use of resources. It encompasses decision-making capabilities, potential improvements and the efficient allocation of resources within hospitals. In the healthcare sector, efficiency studies have mainly used Data Envelopment Analysis (DEA), which evaluates a group of decision-making units (DMUs), with efficient units receiving a performance score of one. Most hospital efficiency research has been conducted at the microlevel, focusing on specific departments or services within each healthcare facility [1,4,5]. These studies aim to identify the efficiency of different hospital units or services, often using DEA. This methodology has proved crucial in assessing operational efficiency in a variety of healthcare settings, including emergency departments, ambulatory care and intensive care units [5]. Despite growing interest in this field, there is still a relative dearth of comprehensive economic analyses encompassing the overall efficiency of public hospitals during such crises. Recent literature suggests integrating DEA with Multi-Criteria Decision Analysis (MCDA) to improve the assessment of hospital efficiency. MCDA facilitates a more nuanced assessment by incorporating multiple criteria and considering the preferences of decision-makers [6]. This approach is particularly relevant in healthcare, where the importance of various inputs and outputs can vary considerably, reflecting hospital management priorities. Such analytical tools help ensure that efficiency assessments do not disproportionately favor less critical aspects of hospital operations to the detriment of more crucial ones.

Public hospitals in Greece, during the COVID-19 epidemic in 2020, represent a significant area of study in healthcare management, particularly with regard to their operational efficiency. There is a notable gap in research on the efficiency of these hospitals during such a critical period. The aim of this research is to fill this gap by improving existing methodologies and incorporating additional criteria that influence efficiency but are not easily quantifiable.

The objectives of this paper are as follows:

1. To develop a Data Envelopment Analysis (DEA) model to assess the efficiency of public hospitals.
2. To determine the level of technical efficiency of these hospitals using the DEA models.
3. To develop an Analytical Hierarchy Process (AHP) model for evaluating hospital efficiency.
4. To analyze the efficiency levels of public hospitals in Greece.
5. To compare the results from both the DEA and AHP models.

The study is organized into five main sections. After the introduction, which contextualizes the impact of COVID-19 on Greek public hospitals, the second section details the methodologies used to assess effectiveness, specifically Data Envelopment Analysis (DEA) and the Analytical Hierarchy Process (AHP). The third section outlines the data collection methodology via a questionnaire distributed to administrative staff at 38 hospitals, targeting a direct assessment of operational efficiency. The fourth section analyzes the results obtained, comparing performance across the DEA and AHP models, and discusses the variability of efficiency scores between hospitals. Finally, the last section summarizes the main findings, highlighting successes and challenges, and recommends the integration of the DEA and AHP approaches to improve future evaluations in times of crisis.

This study innovates by combining the DEA and AHP models for a multidimensional analysis of hospital efficiency during a health crisis, filling a notable gap in the literature. The main contribution lies in the integration of qualitative and quantitative criteria, offering an evaluation framework adaptable to the strategic priorities of public health decision-makers.

## 2. Literature Review

### 2.1. Efficiency Assessment Approaches

Efficiency is defined as the relationship between results produced and resources used and is a key metric for assessing the operational performance of organizations and nations [7]. This metric captures the essence of the effective use of resources to produce desired results [8]. Increased efficiency generally requires the adoption of innovative technologies and the implementation of various procedural or systemic changes. Greater efficiency is characterized by the ability to produce more results with fewer resources. Achieving the maximum possible output for each input unit means optimal efficiency, a goal often difficult to reach without technological advances or significant process modifications. In the absence of these interventions, increasing efficiency remains a major challenge. In the field of performance measurement, efficiency is crucial and can be analyzed using parametric or non-parametric methods [9]. Parametric approaches involve defining a production function and examining the impact of different variables on the result. These methods, which include factor analysis, regression analysis and the stochastic frontier approach, help quantify the effects of various inputs on a system’s outputs [6].

In contrast, non-parametric methods evaluate efficiency without a predetermined production function. Techniques such as Data Envelopment Analysis, the Hierarchical Analytic Process, backward error propagation and artificial neural networks are used in this framework [9]. These approaches exploit linear programming and other mathematical models to assess performance, offering a flexible and often more comprehensive analysis of efficiency without the constraints of predefined functional forms.

The production function is crucial for assessing the operational performance of an organization or nation and is fundamentally defined by the relationship between inputs and outputs [10]. This function reflects the current state of accessible technology within a sector and is directly linked to its economic efficiency. For example, a healthcare facility can be considered technically inefficient if it operates below its capacity frontier, indicating a gap between potential and actual performance. To improve efficiency, the integration of innovative technologies and the implementation of diversified modifications are essential. These initiatives aim to raise operational standards by optimizing the use of available resources.

Performance in an organizational context is often characterized by a combination of effectiveness and efficiency. While effectiveness focuses on the optimal allocation of resources—doing things right—efficiency is concerned with achieving predefined objectives—doing the right things [11]. Efficiency therefore concerns the way in which resources are distributed between alternative uses, seeking to minimize inputs to achieve desired outputs. It is not simply a measure of market success but an indicator of operational excellence and the judicious use of resources. This includes aspects such as decision-making, potential for improvement and benchmarking of resource allocation [9].

Efficiency, as in other sectors, plays a significant role in areas such as public health in times of crisis. It becomes particularly relevant in scenarios where strategic objectives, such as public health emergency management, are paramount [5]. The effectiveness of these efforts is reflected in the ability of health services to adapt and respond to community needs, thereby improving overall social and economic resilience.

To measure efficiency, two predominant methodologies are used: DEA and the AHP. These two approaches offer distinct advantages in assessing the “rightness” of operational activities, providing frameworks that help to understand and improve the efficiency of the organizations studied [5].

### 2.2. DEA

Data Envelopment Analysis is a non-parametric method using linear programming to evaluate the efficiency of organizations. This method defines an efficiency frontier, or envelope, which encompasses all the organizations analyzed [10]. The efficiency of each organization is measured according to its proximity to this boundary [12]. DEA evaluates efficiency by comparing each decision-making unit (DMU), in this case hospitals, with the others in the sample. It thus establishes a “best practice” boundary, representing the highest possible level of output for each level of input. This model does not assume a specific functional form for production and allows the analysis of scenarios with multiple outputs. Estimates of technical efficiency are obtained through these comparisons, focusing on actually observed performance rather than theoretical maxima [10].

The original DEA model (CCR model) proposed by Charnes, Cooper and Rhodes [13] maintains that efficiency is achieved by maximizing the output–input ratio. This model assumes constant returns to scale, implying that proportional increases in inputs will produce proportional increases in outputs. In contrast, the BCC model, introduced by Banker, Charnes and Cooper in 1984, incorporates variable returns to scale by recognizing potential constraints on input utilization [5].

The ability of DEA to handle multiple inputs and outputs simultaneously, without requiring assumptions about data distribution, provides a robust framework for efficiency analysis [6]. This method effectively handles the complexities resulting from different scales of measurement and avoids subjective bias by relying on objective weighting of data during the optimization process [14].

As a well-established empirical methodology, DEA is widely recognized for assessing efficiency and productivity in a variety of fields, including healthcare [6]. It has proved particularly useful in contexts where multiple service outputs require careful evaluation, as in the public health sector during the pandemic.

### 2.3. AHP

The Analytic Hierarchical Process is a structured method used to organize and analyze complex decisions, based on mathematical and psychological principles. This method helps decision-makers establish priority scales by comparing elements in pairs, thus determining the importance of various criteria in multi-criteria decision-making contexts [5]. The process involves capturing the weights of criteria and the advantages of alternatives through pairwise comparisons and then organizing these criteria in a hierarchical manner [15]. In the AHP method, a hierarchy is typically structured in three levels: the top level represents the objectives, the middle level contains the criteria, and the bottom level accommodates the alternatives. At each level, only a limited number of elements (typically 3 to 5) are considered to maintain focus and management. The importance of each criterion is expressed in a matrix that compares them, using a scale of 1 to 9 to assess their relative importance [16].

The methodology adopted is based on a linear additive model in which weights at all levels are determined on the basis of full pairwise comparisons of criteria. This quantitative approach enables decision-making alternatives to be ranked according to their ability to meet the established criteria, reflecting the decision-maker’s priorities [16].

Thanks to its simple structure, ease of implementation and adaptable framework, the AHP has been widely studied and applied in various fields. It is used as a decision-support tool in many sectors such as commerce, industry, healthcare, government and education, demonstrating its versatility and effectiveness in aiding decision-making processes [5].

### 2.4. Utilization of Combined Models for Assessing Efficiency

The combination of the AHP and DEA is less commonly represented but offers promising results when used. Typically, the AHP is integrated with various methodologies, including mathematical programming, SWOT analysis, DEA and Quality Function Deployment (QFD). The integration of the AHP improves decision-making processes, offering a more realistic assessment compared to the use of DEA alone.

A noteworthy application of the combined AHP and DEA methodology was reported in a case involving the relocation of several government agencies in Japanese cities. In this example, the AHP facilitated the determination of the relative importance of various criteria and attributes, which informed the DEA analysis [4]. Another study proposed a combined AHP-DEA approach to facility design problems, using a computer-aided layout design tool called Spiral to create several alternative layouts [17]. In addition, a similar integrative approach was used to measure the relative efficiency of slightly inhomogeneous decision-making units [18].

Relevant research introduced an innovative improvement to this methodological fusion by proposing an additional ranking mechanism for efficient units—those achieving an AED score of 1 [5]. Using the AHP, the researchers provided a more detailed ranking within this group of top-performing units. The research mentioned developed two original models for classifying these effective units using multi-criteria decision-making techniques, specifically goal-based programming and analytic hierarchy [19]. These models demonstrate the potential of combining these analytical tools to refine the evaluation and classification processes in data analysis, thereby improving the depth and usefulness of efficiency evaluations.

The authors of the present work identify a lack of scientific works using both DEA and AHP methods to evaluate Greek healthcare institutions as DMUs during crisis periods. In this study, the efficiency of public hospitals in Greece is examined for the period of the COVID-19 epidemic using DEA and AHP models separately and finally comparing the results of the two approaches.

## 3. Data Sources and Methodology

The DEA (BCC-O, BCC-I, CCR-O, CCR-I) and AHP models were used to assess the efficiency of public hospitals in Greece during the COVID-19 epidemic, focusing on 38 public hospitals that had implemented additional health services. These hospitals represent the largest health institutions in the country, which were consequently heavily impacted and operationally stressed during the pandemic, thus providing a representative sample for the study. The present study used an online survey tool to collect data, which was distributed by e-mail to the administrative staff of these hospitals. This questionnaire comprised seven (7) items, which were presented in a pairwise comparison on a nine (9)-point scale (1–9). Participants were requested to evaluate which item in every competing pair was more important for the effectiveness and the efficiency of the hospital they were employed, with possible answers ranging from one (1), denoting neutrality, to nine (9), suggesting maximum importance for an item. A total of 98 responses were collected over three periods, but only 50 participants from 38 public hospitals provided complete questionnaires suitable for detailed effectiveness analysis. Statistical power was calculated using Python’s statsmodels library, specifically its TTestIndPower class, which is part of the statsmodels.stats.powermodule, to ensure that the sample size of 50 participants was adequate. By increasing the expected effect size to d = 0.6, statistical power attained a value of 84.4%, thereby exceeding the standard 80% threshold often recommended in the literature [20]. The procedure involved acquiring an Institutional Review Board Statement from the University of West Macedonia (permit number 219 30 May 2024) and ensuring informed consent from participants before commencing the research process. All participants were fully informed about the research’s scope, and their anonymity was assured, followed by a detailed explanation of the way in which participants’ data will be used. Last but not least, participants were informed that there were no risks involved in participating. Based on the quantitative results obtained from the feedback of the 50 participants, who engaged inthepairwise comparison and rating of seven (7) factors related to hospital effectiveness and efficiency, a total of 7 weighted items emerged. These factors were adopted both as inputs and outputs of the DEA analyses as well as weighted criteria for the AHP model. The data for these elements (output, input, weighted criteria) were obtained from the official health facilities’ performance metrics database of the Greek Ministry of Health after applying for permission. The correlation of the ranks of all five (5) models (four DEA and one AHP) was further investigated by correlation analysis using Spearman’s correlation coefficient. For the grammar, spelling and syntax correction of the present document in the English language, the Large Language Model was used.

### Development of the DEA Model

For the development of the DEA model, the Open Source DEA (OSDEA-GUI v.3.2) software tool was employed [21]. This analysis incorporated the fundamental DEA models, the BCC (Banker, Charnes and Cooper) and CCR (Charnes, Cooper and Rhodes) models. These models analyzed input-oriented variables with variable returns to scale (VRS) and an output-oriented model with constant returns to scale (CRS). The application of the CCR model yielded a measure of integrated efficiency, known as technical efficiency (TE) [22]. This evaluation helps in understanding how well hospitals managed their resources relative to the outputs they achieved, crucial during the resource-strain periods of the pandemic. Efficiency in the CCR model is mathematically expressed by the following functions, adapted as follows [23]:

(1) $$Max\frac{\sum_{r=1}^{n}\;\left(u_{rb}\right)\left(y_{rb}\right)}{\sum_{k=1}^{m}\;\left(v_{kb}\right)\left(x_{kb}\right)}$$

with the prerequisite that

(2) $$\begin{array}{c} \frac{\sum_{r=1}^{n}\;\left(u_{rb}\right)\left(y_{rj}\right)}{\sum_{k=1}^{m}\;\left(v_{kb}\right)\left(x_{kj}\right)}\leq 1\;for\;each\;unitj\\ u_{rb},v_{kb}\geq \varepsilon \;for\;each\;unitr,k \end{array}$$

$$\begin{array}{l} y_{rj}=out\;put\;vector\;r\;built\;with\;unit\;j\\ x_{kj}=input\;vector\;k\;built\;with\;unit\;j\\ u_{r}=output\;weight\;r\;on\;basic\;unit\;b\\ v_{i}=input\;weight\;I\;on\;basic\;unit\;b\\ j=number\;of\;DMU\\ r=number\;of\;outputs\\ k=number\;of\;inputs\\ \varepsilon =small\;positive\;number \end{array}$$

The BCC model focuses on assessing the pure technical efficiency (PTE) of DMUs [22]. In our study, these DMUs are represented by public hospitals. The performance evaluation of these units via the BCC model is encapsulated by the following formula:

(3) $$\underset{u,v,\omega }{max}\theta_{b}=\sum_{r=1}^{s}u_{r}\left(y_{rjb}\right)+\omega$$

with the prerequisite that

(4) $$\sum_{i=1}^{m}v_{i}\left(x_{ijb}\right)=1$$

(5) $$\sum_{r=1}^{s}u_{r}\left(y_{rj}\right)-\sum_{i=1}^{m}v_{i}\left(x_{ij}\right)+\omega \leq 0$$

$\begin{array}{l} u_{r}\geq \varepsilon \\ v_{i}\geq \varepsilon \\ r=1,2,3,\ldots ,s\\ i=1,2,3,\ldots ,m\\ j=1,2,3,\ldots ,n\\ \omega =free \end{array}$

The ratio between the two models represents total technical efficiency, which comprisestechnical efficiency due to business volume (technical efficiency according to the CRS model) and pure technical efficiency (technical efficiency according to the BCC model), and it is calculated as follows [23].

(6) $$SE=\frac{{TE}_{CRS}}{{TE}_{BCC}}$$

The basis (lowest level) of the tree structure is composed of the 38 public hospitals of our study, which comprise the DMUs. For the DEA model, the following inputs and outputs were included:

Inputs

- Input 1: Number of developed beds
- Input 2: Personnel cases
- Input 3: Medical supplies

Outputs

- Output 1: Number of patients treated
- Output 2: Total days of hospitalization
- Output 3: Total number of laboratory examinations
- Output 4: Total number of surgical procedures

## 4. Development of the AHP Model

**Figure 1:**
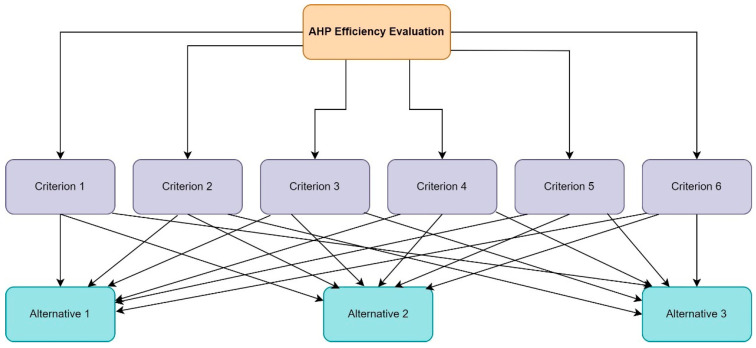
Illustration of the AHP model structure with the inclusion of basic criteria. On the base of the model, the alternatives are established.

The relevant literature suggests the adoption of a common approach to problem structuring, involving a three-level hierarchy of objectives, criteria and alternatives. As illustrated in Figure 1, objectives are at the top level, criteria occupy the middle level, and alternatives are at the bottom. The main function of this hierarchical structure is to facilitate the determination of the relative importance of each element within a given level while taking due account of the influence of elements at lower levels [24].

For the purposes of strength comparison for each structure of the AHP approach, the following formula is adopted:

(7) $$V(x)=W_{1}X_{1}+W_{2}X_{2}+\ldots +W_{m}X_{m}$$

$$W_{i}\text{—}\mathrm{weight}\;\mathrm{that}\;\mathrm{belongs}\;\mathrm{to}\;\mathrm{the}\;i\text{-}\mathrm{th}\;\mathrm{criterion}\;\mathrm{and}\;\mathrm{measures}\;\mathrm{the}\;\mathrm{importance}\;\mathrm{of}\;\mathrm{this}\;\mathrm{criterion}$$

$$x_{i}\text{—}\mathrm{the}\;\mathrm{value}\;\mathrm{of}\;\mathrm{the}\;i\text{-}\mathrm{th}\;\mathrm{criterion}\;\mathrm{for}\;\mathrm{the}\;\mathrm{alternative}\;X$$

The function’s value for a given alternative (x) is computed as the sum of the products $W_{i}\times X_{i}$, where $W_{i}$ is the weight assigned to the i-th criterion, indicating its importance, and $x_{i}$ is the value of the i-th criterion for alternative x. This necessitates the evaluation of both the weights and the alternatives for each criterion. The value function $V\left(x\right)$ quantifies the desirability of alternative x. Alternatives are ranked using this value function, with alternative x deemed more desirable than alternative y when $V(x)>V\left(y\right)$ [25].

To develop the AHP model, the Super Decisions computer program was used. This software, in addition to open-source DEA software—OSDEA-GUI (v. 0.2, is used for AHP modeling. Super Decisions supports decision-making not only through the AHP but also through the Analytic Network Process (ANP). Both methodologies are based on the same fundamental prioritization process, which derives priorities from pairwise comparisons [26].

The first step is to define the decision problem, here represented by the evaluation of the efficiency of public hospitals within a selected sample of 38 (alternative) units. Subsequent steps involve structuring a hierarchy as illustrated in Figure 1. This hierarchy typically features a tree-like structure where higher-level attributes depend on those of lower levels, enabling decision-makers to decompose a complex multi-criteria decision problem into fundamental components. At the highest level, the objective is the evaluation of effectiveness by the AHP; the intermediate level comprises the criteria; the lowest level includes the alternatives, in this study represented by public hospitals.

The development of the AHP model involved establishing seven (7)criteria of public hospitals, which are exactly matched with the outputs and inputs established in the DEA models and for which the participants of the quantitative research were requested to declare preference in pairwise comparison in regard to each compared item’s importance for achieving efficiency of operations in a public hospital unit. The results obtained were entered as weights during the AHP pairwise judgment process in the relevant interface of the Super Decisions software (v. 3.2) thus, the AHP model was developed for direct comparison with DEA models.

AHP criteria:

- Input 1: Number of developed beds
- Input 2: Personnel cases
- Input 3: Medical supplies

Outputs:

- Output 1: Number of patients treated
- Output 2: Total days of hospitalization
- Output 3: Total number of laboratory examinations
- Output 4: Total number of surgical procedures

## 5. Results

### 5.1. CCR Model and BCC Model Results

The analysis of the results of DEA to assess the efficiency and performance of 38 Greek hospitals during the COVID-19 pandemic in 2020 (Table 1) highlights the operational challenges and successes of these healthcare facilities during times of crisis. The results of input- and output-oriented BCC models, which take into account variable returns to scale (VRS), offer a detailed view of how these hospitals managed resources and delivered services. In the BCC models, the average efficiency scores are 83% for the output-oriented model and 78% for the input-oriented model. These averages indicate a commendable level of operational efficiency among the hospitals. The slightly higher average in the output-oriented model suggests a certain pre-eminence in maximizing services produced given inputs. This indicates that hospitals were somewhat better at improving patient care delivery than at managing their resources.

In contrast, the results of the CCR models, which assume constant returns to scale (CRS), present a striking contrast, with average efficiency scores of 65% for both input- and output-oriented models. This reveals less optimal performance, indicating notable scale inefficiencies potentially exacerbated by the varied pressures of the pandemic.

Several hospitals achieved full efficiency (score of 1) in different models. However, a significant gap is evident between the results of the VRS and CRS models. The uniformity of lower efficiency scores in the CCR models implies that many hospitals, although performing well under variable returns, do not maintain this efficiency under constant returns. To quantify these findings, according to the BCC models, 31.58% of hospitals achieved total efficiency in the output-oriented model and 28.95% in the input-oriented model. In the CCR models, 18.42% of hospitals were judged to be totally efficient in both the output- and input-oriented models.

### 5.2. AHP Model Analysis Results

Analysis of the results of the AHP model reveals the efficiency and effectiveness of the Greek hospitals during the COVID-19 outbreak in 2020 in managing the health crisis. According to the results presented in Table 2, efficiency scores vary considerably, reflecting diversity in the management and performance of different hospitals (DMUs).

The highest score observed is 1.00 for DMU4, indicating optimal performance according to the criteria established in the AHP model. This suggests that DMU4 made exemplary use of its resources to respond effectively to the demands imposed by the pandemic. In contrast, the lowest score was 0.486 for DMU37, revealing shortcomings in management efficiency or in the ability to meet critical needs during the examined period.

The distribution of scores shows that two hospitals (5%) maintain a score below 0.50, reflecting very low efficiency. Around 32% of the units (12 hospitals) present scores between 0.50 and 0.69, indicating moderate performance. The majority of hospitals, 21 out of 38 (55%), are within an efficiency range of 0.70 to 0.89, demonstrating effective management and successful adaptation to the challenging conditions of the pandemic. Five hospitals (13%) achieved an efficiency of 0.90 to 1.00, illustrating an excellent ability to maximize performance despite the challenges imposed by COVID-19.

The average efficiency calculated for the total of DMUs is 0.768, reflecting a fairly high level of performance overall but also underlining the importance of continuous improvement for those in the lower brackets.

### 5.3. Comparison of the Obtained Results from the AHP and DEA Model Runs

To evaluate the results of both methods, a correlation analysis using Spearman’s correlation (Table 3) coefficient as a non-parametric measure of rank correlation (statistical dependence between the rankings of two variables) was carried out [27,28]. This assesses how the relationship between two variables can be described using a monotonic function, and thus, efficiency ranks of all models were utilized as input variables.

Analysis of the correlations between the efficiency rankings of the DEA models (BCC and CCR, both inlet and outlet oriented) and those obtained by the AHP method shows divergent results. According to Spearman’s coefficients, a significant correlation exists between the results of the DEA models, particularly strong between the output and input orientations of the BCC model (ρ = 0.974), indicating a strong monotonic dependency between these variables. This observation also extends between the results of the CCR models (ρ = 1.000), underlining a perfect correlation.

However, the correlation between the efficiency ranks of the DEA models and those of the AHP is much weaker. For example, the Spearman coefficient between the AHP and the output-oriented BCC model is 0.226, with a two-tailed significance of 0.167, suggesting a lack of significant statistical correlation. Similarly, correlations between the AHP and the other DEA models are weak (ρ ≤ 0.178) and insignificant (*p* ≥ 0.277).

These results suggest that, although the DEA methods reflect consistent aspects of efficacy within the methods themselves, the AHP method, perhaps incorporating qualitative and subjective criteria in its assessments, does not appear aligned with the purely quantitative DEA measures. We can conclude that the null hypothesis, postulating the absence of correlation between DEA and AHP efficacy ranks, cannot be rejected.

## 6. Discussion

The present research is essential as it addresses the operational performance of hospitals under unprecedented pressure from the increased demands of healthcare systems [29,30]. In line with previous methodologies used in studies by Hannan et al. (1981) [31] and Odynocki (1983) [32], this research incorporates a multi-criteria approach to evaluate hospitals in a more nuanced way.

In all DEA models (BCC and CCR), hospitals displayed efficiency scores that reflected diversity in resource management and service delivery. Notably, the BCC models demonstrated an average efficiency of 83% for the outcome orientation and 78% for the input orientation, indicating relatively efficient management of available resources. In contrast, CCR models revealed notable inefficiencies with average scores of 65%, highlighting scale inefficiencies possibly exacerbated by the varied pressures of the pandemic [33].

The use of the AHP enabled a more refined analysis, revealing that some hospital units made optimal use of their resources to respond effectively to the demands of the health crisis. However, the AHP results, while providing a valuable assessment of individual hospital performance, show little correlation with the results of the DEA models, indicating that the qualitative criteria and subjective preferences incorporated in the AHP do not always align with the pure quantitative measures of DEA.

The discrepancy between AHP and DEA results is consistent with previous work by Longo and Masella (2002) [34] and Chang (2006) [35], where the AHP was applied to evaluate alternative organizational processes in different operating theaters and the quality of services in a healthcare facility. Criteria such as patient care, nursing staff attitude and management, judged by the authors in collaboration with nursing staff, demonstrated the importance of considering both qualitative and quantitative aspects in assessing effectiveness.

Although the DEA and AHP models offer valuable insights into effectiveness, their integration enables a more robust assessment tailored to the complexities of the healthcare sector. This research highlights the need for further development of combined methodologies to improve hospitals’ ability to respond in times of crisis, taking into account both operational performance and overall economic impacts. These results should encourage wider acceptance of formalized methods among medical practitioners, in line with the recommendations of Ahsan and Bartema (2004) [20], for shared and more effective decision-making in the public health context.

## 7. Conclusions

In this paper, the operational efficiency of 38 Greek hospitals during the COVID-19 epidemic in 2020 is examined through a detailed analysis using DEAmodels (BCC-O, BCC-I, CCR-O, CCR-I) and the AHP model with weighted criteria thatwerechosenbased on the quantitative results obtained from the feedback of 50 participants from 38 public hospitals in Greece who engaged inthepairwise comparison and rating of a total of seven factors related to hospital effectiveness and efficiency. These factors were adopted both as inputs and outputs of the DEA analyses as well as weighted criteria for the AHP model. This multidimensional approach provides a comprehensive picture of the performance of the decision-making units under study, in a context where hospitals have been under extreme pressure during the health crisis.

The results obtained with the BCC models, which take into account variable returns to scale (VRS), show average efficiency scores of 83% for the result-oriented model (BCC-O) and 78% for the input-oriented model (BCC-I). These scores testify to theremarkable ability of hospitals to maximize outputs given inputs, suggesting a relatively efficient management of available resources.

In contrast, the CCR models, which assume constant returns to scale (CRS), show uniformly lower efficiency scores of 65% for both orientations. This observation highlights notable scale inefficiencies, exacerbated by the various pressures of the pandemic, revealing that although hospitals performed well under variable returns, their efficiency is not maintained under constant returns.

The application of the AHP model enabled a more nuanced analysis by integrating multiple criteria and decision-makers’ preferences. This method reveals that some hospital units were able to make optimal use of their resources to respond effectively to the demands imposed by the health crisis. Nevertheless, the AHP results, while providing a valuable assessment of individual hospital performance, show little correlation with the results of the DEA models, indicating that the qualitative criteria and subjective preferences incorporated in the AHP do not always align with the purely quantitative DEA measures.

This study highlights the importance of an integrated approach that combines the quantitative analyses of DEA with the qualitative assessments of the AHP to provide a comprehensive insight into hospital efficiency. The results demonstrate not only the ability of hospitals to manage direct healthcare impacts during the pandemic but also their role in supporting broader entrepreneurial goals within the healthcare sector, such as the rapid expansion of digital healthcare services and innovations in patient care management.

To successfully incorporate the integration of a combined DEA and AHP methodology as a methodological synthesis, researchers could develop their systemic approach as follows: initially, DEA could be used to assess the technical efficiency of units, identifying best practices among public hospitals; subsequently, the AHP could be applied to weight efficiency criteria relating to inputs and outputs, enabling resources to be prioritized on the basis of preferences defined by decision-makers.

The evidence indicates that public hospitals succeeded in maximizing their services during a period characterized by resource scarcity, through adaptive management of inputs and processes. During future health crises, it is essential to strengthen hospital resources and adopt effective organizational practices. The DEA and AHP models revealed that the best-performing hospitals rely on a robust organizational structure and adequately trained staff, key elements for optimizing resilience in health crisis contexts.

This research enriches the existing body of scientific literature on evaluating hospital efficiency and proposes improved methodologies for analyzing performance in times of crisis. Prospects for future research will include the exploration of combined methods to further refine effectiveness assessment, essential for strategic decision-making in the management of healthcare services in times of crisis.

A novelty of this study is the combination of DEA and AHP models to assess the efficiency of Greek public hospitals during the COVID-19 pandemic as a combined analytical approach to added hospital efficiency during health crises. A limitation lies in the use of self-reported data for the definition of the AHP criteria with only 50 complete responses being returned, which may affect the generalizability of results. In theoretical terms, the present study enriches the relevant literature by integrating qualitative criteria into the assessment of efficiency for healthcare institutions. In practical terms, it also provides decision-makers with an analytical framework for optimizing resource management in situations of crisis. For future research, a longitudinal approach could examine the evolution of hospital efficiency over time and incorporate other quantitative methodologies to reinforce the robustness of the results.

## Figures and Tables

**Table 1:** Tabulation of the results for both the BCC and the CCR models (output and input oriented) **.

	BCC—O		BCC—I		CCR—O		CCR—I	
DMU Names	Objective Value	Efficient	Objective Value	Efficient	Objective Value	Efficient	Objective Value	Efficient
DMU1	0.901		0.82		0.543		0.543	
DMU2	1	Yes	1	Yes	1	Yes	1	Yes
DMU3	0.788		0.776		0.617		0.617	
DMU4	1		1	Yes	1	Yes	1	Yes
DMU5	0.912		0.917		0.875		0.875	
DMU6	0.591		0.432		0.424		0.424	
DMU7	1	Yes	1	Yes	1	Yes	1	Yes
DMU8	0.899		0.882		0.783		0.783	
DMU9	0.702		0.51		0.477		0.477	
DMU10	1	Yes	1	Yes	1	Yes	1	Yes
DMU11	0.561		0.421		0.306		0.306	
DMU12	1		1		0.822		0.822	
DMU13	1	Yes	1		0.801		0.801	
DMU14	0.855		0.851		0.681		0.681	
DMU15	0.742		0.73		0.637		0.637	
DMU16	0.781		0.726		0.531		0.531	
DMU17	1	Yes	1	Yes	0.664		0.664	
DMU18	1	Yes	1	Yes	1	Yes	1	Yes
DMU19	0.424		0.085		0.084		0.084	
DMU20	1	Yes	1	Yes	1	Yes	1	Yes
DMU21	0.779	Yes	0.323		0.241		0.241	
DMU22	1	Yes	1	Yes	0.116		0.116	
DMU23	0.855	Yes	0.829		0.561		0.561	
DMU24	1	Yes	1	Yes	0.806		0.806	
DMU25	1	Yes	1	Yes	0.835		0.835	
DMU26	0.751		0.733		0.622		0.622	
DMU27	1	Yes	1	Yes	0.826		0.826	
DMU28	1	Yes	1	Yes	0.507		0.507	
DMU29	0.678		0.488		0.406		0.406	
DMU30	0.925		0.922		0.774		0.774	
DMU31	0.623		0.6		0.55		0.55	
DMU32	0.724		0.711		0.617		0.617	
DMU33	0.844		0.838		0.714		0.714	
DMU34	0.741		0.724		0.621		0.621	
DMU35	0.622		0.606		0.519		0.519	
DMU36	0.577		0.547		0.502		0.502	
DMU37	0.847		0.838		0.727		0.727	
DMU38	0.448		0.413		0.388		0.388	
Average	0.834		0.789		0.654		0.658	

The Banker, Charnes and Cooper model, called BCC, was first introduced in 1984 to introduce variable returns to scale (the CCR model only assumed constant RTS). The only difference with the CCR model is the convexity constraint e*Lambdas = 1/or uoin the multiplier form. The Charnes, Cooper and Rhodes model, called CCR, was first introduced in 1978 and assumes constant RTS. ** In this table, only units with a score of 1 are marked as fully efficient, as per DEA standards. Scores below 1 indicate that these units are less efficient compared to fully efficient hospitals.

**Table 2:** Results of the AHP model analysis.

DMU Names	Value
1DMU1	0.626
2DMU2	0.703
3DMU3	0.684
4DMU4	1.000
5DMU5	0.663
6DMU6	0.736
7DMU7	0.752
8DMU8	0.638
9DMU9	0.712
10DMU10	0.801
11DMU11	0.734
12DMU12	0.810
13DMU13	0.829
14DMU14	0.829
15DMU15	0.747
16DMU16	0.782
17DMU17	0.842
18DMU18	0.759
19DMU19	0.814
20DMU20	0.810
21DMU21	0.849
22DMU22	0.791
23DMU23	0.757
24DMU24	0.853
25DMU25	0.792
26DMU26	0.821
27DMU27	0.866
28DMU28	0.821
29DMU29	0.961
30DMU30	0.795
31DMU31	0.861
32DMU32	0.855
33DMU33	0.623
34DMU34	0.957
35DMU35	0.665
36DMU36	0.524
37DMU37	0.486
38DMU38	0.605
Average	0.767

**Table 3:** Spearman correlation.

Correlations							
			BCC Output Oriented	BCC Input Oriented	CCR Output Oriented	CCR Input Oriented	AHP Weighted Criteria
Spearman’s rho	BCC Output Oriented	Correlation Coefficient	1.000	0.974 **	0.745 **	0.745 **	0.226
		Sig. (2-tailed)	.	0.000	0.000	0.000	0.167
		N	39	39	39	39	39
	BCC Input Oriented	Correlation Coefficient	0.974 **	1.000	0.790 **	0.790 **	0.178
		Sig. (2-tailed)	0.000	.	0.000	0.000	0.277
		N	39	39	39	39	39
	CCR Output Oriented	Correlation Coefficient	0.745 **	0,790 **	1.000	1.000 **	0.088
		Sig. (2-tailed)	0.000	0.000	.	.	0.593
		N	39	39	39	39	39
	CCR Input Oriented	Correlation Coefficient	0.745 **	0.790 **	1.000 **	1.000	0.088
		Sig. (2-tailed)	0.000	0.000	.	.	0.593
		N	39	39	39	39	39
	AHP Weighted Criteria	Correlation Coefficient	0.226	0.178	0.088	0.088	1.000
		Sig. (2-tailed)	0.167	0.277	0.593	0.593	.
		N	39	39	39	39	39

** Correlation is significant at the 0.01 level (two-tailed).

## Data Availability

The data used to support the findings of this study are available from the corresponding author upon request.
